# Does a robot’s gaze aversion affect human gaze aversion?

**DOI:** 10.3389/frobt.2023.1127626

**Published:** 2023-06-23

**Authors:** Chinmaya Mishra, Tom Offrede, Susanne Fuchs, Christine Mooshammer, Gabriel Skantze

**Affiliations:** ^1^ Furhat Robotics AB, Stockholm, Sweden; ^2^ Humboldt-Universität zu Berlin, Berlin, Germany; ^3^ Leibniz-Centre General Linguistics (ZAS), Berlin, Germany; ^4^ Division of Speech, Music and Hearing, KTH Royal Institute of Technology, Stockholm, Sweden

**Keywords:** gaze, gaze aversion, human-robot interaction, social robot, gaze control model, gaze behavior, intimacy, topic intimacy

## Abstract

Gaze cues serve an important role in facilitating human conversations and are generally considered to be one of the most important non-verbal cues. Gaze cues are used to manage turn-taking, coordinate joint attention, regulate intimacy, and signal cognitive effort. In particular, it is well established that gaze aversion is used in conversations to avoid prolonged periods of mutual gaze. Given the numerous functions of gaze cues, there has been extensive work on modelling these cues in social robots. Researchers have also tried to identify the impact of robot gaze on human participants. However, the influence of robot gaze behavior on human gaze behavior has been less explored. We conducted a within-subjects user study (N = 33) to verify if a robot’s gaze aversion influenced human gaze aversion behavior. Our results show that participants tend to avert their gaze more when the robot keeps staring at them as compared to when the robot exhibits well-timed gaze aversions. We interpret our findings in terms of intimacy regulation: humans try to compensate for the robot’s lack of gaze aversion.

## 1 Introduction

It is well established that gaze cues are one of the most important non-verbal cues used in Human-Human Interactions (HHI) (Kendon, 1967). Several studies have shown the many roles gaze cues play in facilitating human interactions. When interacting with each other, people use gaze to coordinate joint attention, communicating their focus of attention and perceiving their partner’s focus to follow (Tomasello, 1995). Ho et al. (2015) also showed how people use gaze to manage turn-taking: for instance, gaze directed at or averted from one’s interlocutor can indicate whether a speaker is intending to yield or hold the turn (for example, when making a pause), or when the listener is intending to take the turn.

Given the importance of gaze behavior in HHI, researchers in Human-Robot Interaction (HRI) have tried to emulate human-like gaze behaviors in robots. The main motivation behind such Gaze Control Systems (GCS), or models of gaze behavior, has been to exploit the many functionalities of gaze cues in HHI and realize them in HRI. Moreover, thanks to the sophisticated anthropomorphic design of many of today’s social robots (e.g., Furhat robot (Moubayed et al., 2013) or iCub robot (Metta et al., 2010)), it is possible to model nuanced gaze behaviors with independent eye and head movements. It has been established that robots’ gaze behaviors are recognized and perceived to be intentional by humans (Andrist et al., 2014). Robots’ gaze behaviors have also been found to play an equally important role in HRI as human gaze in HHI (Imai et al., 2002; Yamazaki et al., 2008). Thus, researchers have measured the impact of robots’ gaze behavior on human behavior during HRI. In Schellen et al. (2021) participants were found to become more honest in subsequent trials if the robot looked at them when they were being deceptive. Skantze (2017) and Gillet et al. (2021) observed that robots’ gaze behavior could lead to more participation during group activities. Most of these works have concentrated on human behavior in general, but not the gaze-to-gaze interaction between robots and humans. This then leads to our research question.

•*Does a robot’s gaze behavior have any influence on human gaze behavior in a HRI?*

Answering this question is important because it can help in designing better GCS and interactions in HRI. Even though previous works have shown various ways in which humans perceive and respond to robot gaze behavior, whether there are changes in human gaze behavior as a direct influence of robots’ gaze behavior has remained less explored. Moreover, most of these studies have used head movements instead of eye gaze to model robot gaze behavior, due to physical constraints of the robots used (Andrist et al., 2014; Mehlmann et al., 2014; Nakano et al., 2015). While head orientation is a good approximation of gaze behavior in general, it lacks the rich information ingrained in eye gaze. Additionally, from a motor control perspective, eye gaze is much quicker than head motion and is therefore also more adaptable than moving the head. Thus, we were interested in verifying if subtle gaze cues performed by a robot are perceived by humans and if it had any influence on their own gaze behavior.

In order to verify the impact of robot gaze behavior, we narrowed our focus to gaze aversions for this study. This was mainly motivated by two considerations. First, gaze aversion has been shown to play an important role in human conversations: coordinating turn-taking (Ho et al., 2015), regulating intimacy (Abele, 1986) and signalling cognitive load (Doherty-Sneddon and Phelps, 2005). Secondly, it is an important gaze cue which is relatively easy to perceive and generate during HRI.

In this work, we designed a within-subjects user study to measure if gaze aversion exhibited by a robot has any influence on the gaze aversion behavior of participants. We automated the robot’s gaze using the GCS proposed in Mishra and Skantze (2022) (more details in Section 3) to exhibit time- and context-appropriate gaze aversions. Participants’ gaze was tracked using eye-tracking glasses throughout the interactions. Subjective responses were also collected from the participants after the experiment, using a questionnaire that asked about their impression of the interaction. Our results show that participants avert their gaze more when the robot doesn’t avert its gaze as compared to when it does.

The main contributions of this paper are:

• The first study (to the best of our knowledge) that verified the existence of a direct relationship between robot gaze aversion and human gaze aversion.
• A study design to measure the influence of a robot’s gaze behavior on human gaze behavior.
• An exploratory analysis of the eye gaze data, which pointed towards a potential positive correlation between gaze aversion and topic intimacy of the questions.

## 2 Background

**Gaze aversion** is the act of shifting the gaze away from one’s interaction partner during a conversation. Speakers tend to look away from the listener more often than the other way around during a conversation. This has been thought to help plan the upcoming utterance and avoid distractions (Argyle and Cook, 1976). It has been found that holding mutual gaze significantly increases hesitations and false starts (Beattie, 1981). Speakers process visual information from their interlocutors, produce speech and plan the upcoming speech, all at the same time. Prior studies in HHI have shown that people use gaze aversions to manage cognitive load (Doherty-Sneddon and Phelps, 2005) because averting gaze reduces the load of processing the visual information. Ho et al. (2015) found that speakers signal their desire to retain the current turn, i.e., turn-holding, by averting their gaze and that they begin their turns with averted gaze. Additionally, gaze aversion has been found to have a significant contribution in regulating the intimacy level during a conversation (Abele, 1986). Binetti et al. (2016) found that the amount of time people can look at each other before starting to feel uncomfortable was 3–5 s.

Several studies have modelled gaze aversion behavior in social robots and evaluated their impact. Andrist et al. (2014) collected gaze data from HHI and used that to model human-like gaze aversions on a NAO robot. They found that well-timed gaze aversions led to better management of the conversational floor and the robot being perceived as more thoughtful. Zhong et al. (2019) controlled the robot’s gaze using a set of heuristics and found that users rated the robot to be more responsive. Subjective evaluation of the gaze system in Lala et al. (2019) showed that gaze aversions with fillers were preferred when taking turns. On the other hand, there have been a few studies that included gaze aversions as a sub-component of their GCS, but they did not measure any effects of gaze aversion (Mutlu et al., 2012; Mehlmann et al., 2014; Zhang et al., 2017; Pereira et al., 2019). For example, Mehlmann et al. (2014) looked at the role of turn-taking gaze behaviors as a whole to evaluate their GCS. However, it is important to note that both Mehlmann et al. (2014) and Zhang et al. (2017) used the gaze behavior of participants as feedback to manage the robot’s gaze behaviors. Mehlmann et al. (2014) grounded their architecture on the findings from HHI, whereas Zhang et al. (2017) relied on findings from human-virtual agent interactions.

Although it has been established that humans perceive robot gaze as similar to human gaze in many cases (Yoshikawa et al., 2006; Staudte and Crocker, 2009), it is still important to verify if it holds for different gaze cues and situations in an HRI setting as findings from Admoni et al. (2011) suggest that robot gaze cues are not reflexively perceived in the same way as human gaze cues. Thus, it is crucial to investigate whether a relationship exists between robot gaze behavior and human gaze behavior, how they are related, and what are the implications of such a relationship. For example, if it is known that lack of gaze aversion by a robot makes people uncomfortable, then we might want to include appropriate gaze aversions when designing a robot for therapeutic intervention. On the other hand, we would probably include fewer gaze aversions when designing an interaction where a robot is training employees to face rude customers. To the best of our knowledge, this is the first work that tries to establish a direct relationship between robot gaze aversion and human gaze aversion behavior.

## 3 Automatic gaze aversion using Gaze control systems

To automate the robot’s gaze behavior in this study, we used the GCS proposed in Mishra and Skantze (2022). It is a comprehensive GCS that takes into account a wide array of gaze-regulating factors, such as turn-taking, intimacy, and joint attention. The gaze behavior of the robot is planned for a future rolling time window, by giving priorities to different gaze targets (e.g., users, objects, environment), based on various system events related to speaking/listening states and objects being mentioned or moved. At every time step, the GCS makes use of this plan to decide where the robot should be looking and to better coordinate eye–head movements.

To model gaze aversion, the model processes the gaze plan at every time step to check if the gaze of the robot is planned to be directed at the user for a duration longer than 3–5 s (the preferred mutual gaze duration from HHI (Binetti et al., 2016)). If that is the case, the model inserts intimacy-regulating gaze aversions into the gaze plan. This results in a quick glance away from the user for about 400 ms using the eye gaze only. Additionally, when the robot’s intention is to hold the floor at the beginning of an utterance or at pauses, the GCS also inserts gaze aversions at the appropriate time to model turn-taking and cognitive gaze aversions.

The parameters of the model are either taken from the literature or tuned empirically. This, combined with the novel eye-head coordination, results in a human-like gaze aversion behavior by the robot. In a subjective evaluation of the GCS through a user study, it was found to be preferred over a purely reactive model, and the participants especially found the gaze aversion behavior to be better (Mishra and Skantze, 2022).

## 4 Hypotheses

Abele (1986) found that too much eye gaze directed at an interlocutor would induce discomfort for the speaker and that periodic aversion of gaze would result in a more comfortable interaction. The *Equilibrium Theory* (Argyle and Dean, 1965) also suggests an inverse relationship between gaze directed at and gaze averted, arguing that increased gaze at an interlocutor would be compensated with more gaze aversions by them. While the theory also discusses other factors such as proxemics, we were interested only in the gaze aspect and in verifying if there is an effect of robot gaze on human gaze behavior. Additionally, it is known that while listening, individuals tend to look more at their speaking interlocutors whereas while speaking, they tend to exhibit more gaze aversions (Argyle and Cook, 1976; Cook, 1977; Ho et al., 2015). Thus, if the robot is not averting its gaze during the interaction, we can expect the participant to produce more gaze aversion while speaking, but not necessarily while listening. Based on this, we formulate the following hypotheses.

•**H1** *Lack of gaze aversions by a robot will lead to an increase in gaze aversions by the participants when they are speaking.*
  • ***H1a:*** *Participants will avert their gaze away from the robot longer in the condition when the robot does not avert its gaze away from the participants. (see* Section 5 *).*
  • ***H1b:*** *Participants will look away from the robot more often when the robot exhibits fixed gaze behavior (does not avert its gaze).*

## 5 Study design

To investigate the effect of a robot’s gaze aversion on human gaze aversion, we designed a within-subjects user study with two conditions. In the control condition, the robot constantly directs its gaze towards the participant, without averting it; we call this the *Fixed Gaze (FG)* condition. In the experimental condition (which we call the *Gaze Aversion (GA)* condition), the robot’s gaze is automated using the GCS described in Section 3 which is found to be better at exhibiting gaze aversion behavior in a subjective analysis. While the GCS is capable of coordinating individual eye and head movements, the interaction is designed in such a way that it does not require any head movements by the robot when directing its gaze. This is because the interaction involved mainly intimacy-regulating gaze aversions, which necessitate only a quick glance away from the interlocutor (see Section 3). Hence, the robot’s head movements are not a factor in the study, which is in line with our aim to verify the effect of robot’s eye gaze behavior on human gaze behavior.

### 5.1 Interaction setting

We designed an interview scenario similar to that in Andrist et al. (2014), where the robot asked the participant six questions with increasing levels of intimacy (more details in Subsection 5.2). While Andrist et al. (2014) investigated whether appropriate gaze aversions by the robot would elicit more disclosure, we wanted to verify if gaze aversions by a robot would directly elicit lower gaze aversions by humans, signaling more comfort even with highly intimate questions (which are known to induce discomfort). To make the interaction more conversational and less one-sided, the robot also gave an answer to each question after the participant had answered it. Questions with different levels of intimacy were used in order to vary the level to which the participant might feel the need to avert their gaze.

The robot’s turns were controlled by the researcher using the Wizard-of-Oz (WoZ) approach. The researcher listened to the participant’s responses through a wireless microphone and controlled the robot’s response by selecting one of three options, which resulted in varying flows of the conversation script. On selecting “Robot answer”, the robot would answer the question that was asked to the participant before moving on to ask the next question. The option “User declined to answer” would prompt the robot to acknowledge the user’s choice before moving on to the answer, and then ask the next question. The “User asked to repeat question” option was used to repeat the question. Having a WoZ paradigm enabled the researcher to control the timing of the robot’s turn-taking, resulting in a smooth conversational dynamics. Additionally, it made it possible for the researcher to manage the interaction from a separate room, reducing the influence that the presence of a third-person observer might have on the participants. The robot’s responses were handcrafted to be generic enough to account for most of the answers that participants might provide. They always started with an acknowledgement of the participant’s answer (e.g., “*I appreciate what you say about the weather*”). Then a response was chosen at random from previously created pool of handcrafted answers to the question and appended to the acknowledgement. In cases where the participant did not answer the question, the robot always acknowledged that by using phrases like “*That’s okay*” and then appended a random response from the pool of answers.

An example dialog where the participant answered the question is provided below (R denotes the robot, P denotes a participant).

R: *What do you think about the weather today?*
P: *I think it is perfect. It is neither freezing nor too hot. Just the perfect balance of sunny and cool. I really don’t like if it is too hot or too cold.*
R: *I appreciate what you say about the weather, but honestly, I can’t relate. I never get to go outside. Maybe you didn’t notice, but I don’t have legs. So I never have any idea what the weather is like out in the real world. My dream is to 1 day see the sky. Perhaps my creators will allow me some day.*
R: *What are your views on pop music?*

We used a Furhat robot for the study, which is a humanoid robot head that projects an animated face onto a translucent mask using back-projection and has a mechanical 3-DoF neck. This makes it possible to generate nuanced gaze behavior using both eye and head movements, as well as facial expressions and accurate lip movements (Moubayed et al., 2013). For the experimental condition (Gaze Aversion; GA) the robot was named Robert and for the control condition the robot was named Marty. We wanted to give the impression that the participants were interacting with two distinct robots for each condition, but at the same time, we did not want the robots themselves to have an influence on the interaction. This led to the selection of two faces that were similar to each other from the list of characters already available in the robot. Two male voices were selected from the list of available voices based on how natural they sounded when saying the utterances for the tasks. The participants were not informed about the different gaze behaviors of the two robots.

**FIGURE 1:**
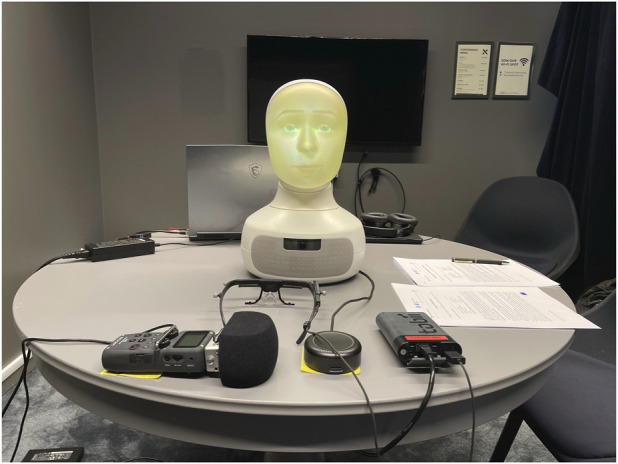
Experimental setup for the interview task.

The experiment was conducted in a closed room while restricting any outside distractions. The participants were alone with the robot during the interactions. Participants were asked to sit in a chair that was placed approximately 60–90 cm in front of the robot. The robot was carefully positioned such that it was almost at eye level and at a comfortable distance for the participants. A Tobii Pro Glasses 2 eye-tracker was used to record the participants’ eye gaze during each interaction. We also recorded the speech of the participants using a Zoom H5 multi-track microphone. A pair of Rode Wireless Go microphone systems was also used to stream the audio from the user to the Wizard. Figure 1 shows an overview of the experimental setup.

### 5.2 Intimacy rating of questions

The questions for the task were selected from Hart et al. (2021) and Kardas et al. (2021), who asked their participants to rate them in terms of sensitivity and intimacy, respectively. In order to account for any influence culture and demography might have on the perceived topic intimacy levels of the questions, an online survey was conducted where residents of Stockholm rated these questions based on their perceived topic intimacy. Participants were recruited using social media forums for Stockholm residents (e.g., Facebook groups, Stockholm SubReddit). Another consideration was to avoid complex questions that would involve a lot of recalling or problem-solving (e.g., “What are your views about gun control?”). The motivation for this is that people are known to avert their gaze when performing a cognitively challenging task (Doherty-Sneddon and Phelps, 2005). We wanted to keep the questions as simple as possible so as to restrict the influence on gaze aversions to just the robot’s gaze behavior and the question’s intimacy level.

A total of 28 questions were selected from the questions in Hart et al. (2021) and Kardas et al. (2021). The participants were asked to rate the questions on how intimate they felt on a 9-point Likert scale ranging from “1: Not intimate at all” to “9: Extremely intimate” (question asked: *Please indicate how intimate you find the following questions (1: not intimate at all; 9: extremely intimate). Please don’t think too much about each one; just follow your intuition about what you consider personal/intimate*). The responses from 148 participants (68 females, 76 males, one non-binary and two undisclosed), aged between 18 and 50 (mean = 29.35, SD = 6.89), were then used to order the questions based on their intimacy values. Using linear mixed models, it was verified that gender, age, nationality and L1 did not influence the intimacy ratings. We selected a total of 12 questions out of them and divided them into two sets with similar intimacy distribution which were used evenly across both conditions (*FG* and *GA*). We tried to select simple questions that would not induce a heavy cognitive load. Table 1 lists the questions and their rated intimacy values from the survey.

**TABLE 1:** Mean intimacy ratings of selected questions used in the study.

Question	Mean	SD	Question set
What do you think about the weather today?	1.192	0.558	1
What are your views on pop music?	1.976	1.372	1
How did you celebrate last Christmas?	3.023	1.758	1
Tell me about a conversation you had with another person earlier today	4.330	2.121	1
For what in your life do you feel most grateful?	5.223	1.917	1
What is one of the more embarrassing moments in your life?	6.538	2.016	1
What did you have for breakfast this morning?	1.823	1.308	2
What season do you like the best? Why?	1.838	1.091	2
Do you have anything planned for later today? What will you do?	3.523	1.779	2
What would constitute a perfect day for you?	4.007	1.827	2
Is there something you’ve dreamed of doing for a long time? Why haven’t you done it?	5.430	2.064	2
Can you describe a time you cried in front of another person?	7.023	1.918	2

### 5.3 Participants

We recorded eye gaze and acoustic data of 33 male participants (sex assigned at birth). The choice for male participants was methodologically and logistically motivated. Firstly, topic intimacy has been found to be perceived differently by people of different genders (Sprague, 1999). Thus, intimacy during the interaction might be affected by the participants’ and robot’s gender. To reduce the influence of this variable (given that it is not a variable of interest in this study), we controlled it by recruiting participants of only one gender.

The participants were recruited using social media, notice boards and the digital recruitment platform Accindi (https://www.accindi.se/). The participants were all residents of Stockholm. The cultural background of participants was not controlled for. Participants’ ages ranged between 21 and 56 (mean = 30.54, SD = ±8.07). They had no hearing or speech impairments, had normal/corrected vision (did not require the use of glasses for face-to-face interactions) and spoke English. They were compensated with a 100SEK gift card on completion of the experiment. The study was approved by the ethics committee of Humboldt-Universität zu Berlin.

### 5.4 Procedure

As described earlier, the study followed a within-subjects design. Each participant interacted with the robot under two conditions; the order of the conditions was randomized. Each set of questions (cf. Table 1) was also counterbalanced across the conditions. The participants were asked to give as much information as they could when answering the questions. However, they were not forced to answer any of the questions. In case they did not feel comfortable answering any questions, the robot acknowledged it and moved on to the next question. The interaction always started with the robot introducing itself before moving on to the questions. The entire experiment took approximately 45 min. The experiment’s procedure can be broken down into the following steps.

•**Step 1:** The participants were informed about the experiment’s procedure, compensation, and data protection, both verbally and in writing. They then provided their written consent to participation.
•**Step 2:** The participants were instructed to speak freely about a prompted topic for about 2 min. This recording was used as the baseline speech measure for participants’ speech before interacting with the robot. The speech data is not discussed in the present work.
•**Step 3:** Next, the participants were asked to put on the eye-tracking glasses, which were then calibrated. After successfully calibrating the glasses, the researcher left the room and initiated the interview task. The robot introduced itself and proceeded with the Q&A.
The researcher kept track of the participant’s responses and timed the robot’s turns with the appropriate response using the wizard buttons. Once the interaction came to an end, the researcher returned to the room for the next steps.
•**Step 4:** The participants were then asked to remove the tracking glasses and were provided with a questionnaire to fill in. The questionnaire had 9-point Likert scale questions about the participant’s perception of the robot and the flow of conversation (see Table 2).
•**Step 5:** Next, they were asked to fill out the Revised NEO Personality Inventory (NEO-PI-R) (Costa Jr and McCrae, 2008), which measures personality traits. They were also asked to take the LexTALE test (Lemhöfer and Broersma, 2012), which indicates their general level of English proficiency, on an iPad. Both of these tasks served as distractor tasks, providing a break between the two interactions and allowing the participants to focus on the second robot with renewed attention.
•**Step 6:** The participants were then asked to speak freely about another prompted topic for about 2 min. This served as the baseline for the second interaction before the participant interacted with the robot (data not discussed here).
•**Step 7:** After recording the free speech, the participants were asked to put on the eye-tracking glasses and the tracker was calibrated again. The researcher left the room and initiated the next interaction. The robot introduced itself again and proceeded with the Q&A.
•**Step 8:** At the end of the interaction, the researcher returned to the room and provided the participants with the last questionnaire. Apart from the 9-point Likert scale questions about the perception of the robot and the conversation flow, the questionnaire also asked about basic demographic details.

**TABLE 2:** Questionnaire used for subjective evaluation.

Dimension	Question
Conversation Flow (**D1**)	My conversation with the robot flowed well
	I was able to understand when the robot wanted me to speak
	I was able to understand when robot wanted to keep speaking
	The robot responded to me at the appropriate time
Human-Likeness (**D2**)	The robot’s face was very human-like
	The robot’s voice was very human-like
	The robot’s behavior was very human-like
	Throughout the conversation, I was very aware that I was talking to a robot
Overall Impression (D3)	I enjoyed talking with the robot
	I felt positively about the robot
	I felt positively about the conversation
	I felt comfortable while talking with the robot

### 5.5 Measurements

In order to test **H1**, we mainly focused on the behavioral measure of gaze behavior of the participants, which was captured using the eye-tracking glasses. Our experiment had one independent variable, the *gaze aversion* of the robot which was manipulated in a within-subjects design (*GA* & *FG* condition). The order of the questions remained the same for both the *GA* and *FG* conditions, i.e., increasing intimacy with each subsequent question.

The Tobii Pro Glasses 2 eye-tracker records a video from the point of view of the participant, and provides the 2D gaze points (i.e., where the eyes are directed in the 2D frame of the video). The videos were recorded at 25fps and the eye-tracker sampled the gaze points at a 50 Hz resolution. Both datasets were synchronized to obtain timestamp vs. 2D gaze point ([*ts*, (*x*, *y*)]) data for each recording, i.e., gaze location per timestamp. We used the Haar-cascade algorithm available in the OpenCV library to detect the face of the robot in the videos and obtain the timestamp vs. bounding box of face ([*ts*, (*X*, *Y*, *H*, *W*)], X and Y - lower left corner of the bounding box, H and W - height and width of the bounding box) data.

**FIGURE 2:**
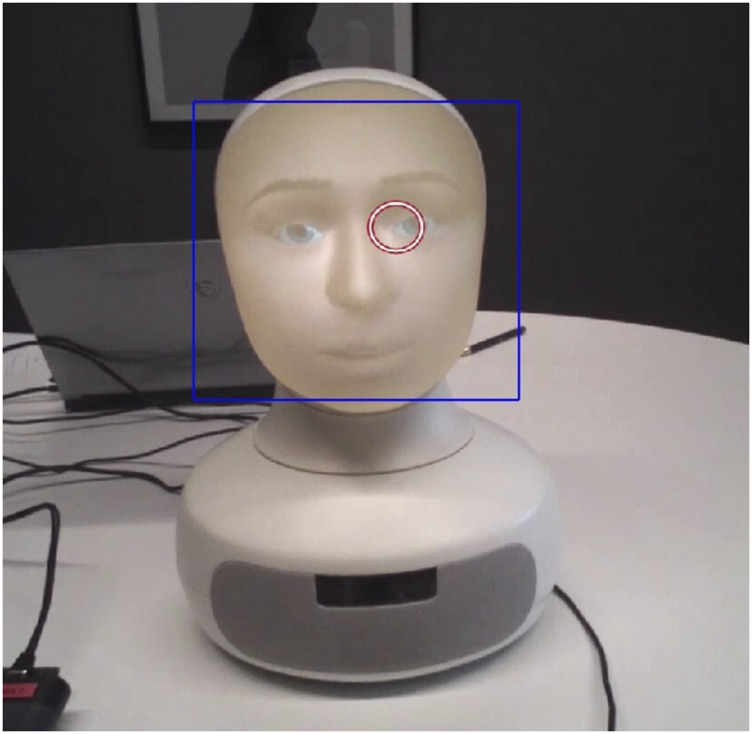
Example of Gaze Aversion detection using the algorithm. Here the gaze point (*x*, *y*) (the red circle) lies within the face’s bounding box [*X*, *Y*, *H*, *W*] (blue rectangle), so it is not a Gaze Aversion.

Gaze Aversion for each time stamp was calculated by verifying if the gaze points (*x*, *y*) were inside the bounding box [*X*, *Y*, *H*, *W*] or not. The parameters for Haar-cascade were manually fine-tuned for each recording to obtain the best fitting bounding boxes for detecting the robot’s face. An example of non-gaze aversion detection using the algorithm can be seen in Figure 2.

The timing information for the robot’s utterances can be obtained from the speech synthesizer. We recorded the robot’s responses and their time information for all interactions. This log was used to extract the participant’s speaking and listening durations. When the robot is speaking, the participant is the listener and *vice versa*. This information was used to extract the gaze aversion of the participant when they were *Speaking* and *Listening*.

For ***H1a***, we used the % of gaze aversion as the metric of overall gaze aversion. Each timestamp where it was possible to detect whether there was a gaze aversion or not was considered as a *gaze event*. We counted the number of gaze aversions (*gaCount*) and the total number of *gaze events* (*geTotal*) over the duration when the participants were *Speaking* and *Listening*. The % of gaze aversion (*ga%*) is then calculated as:

ga%=gaCount/geTotal (1)

For ***H1b***, we identified individual gaze aversion instances, which are the number of times the participants directed their gaze away from the robot. The duration from when participants looked away from the robot until the time they returned their gaze back at the robot was counted as one gaze aversion instance.

We also collected subjective feedback from the participants for both conditions with a questionnaire. The questionnaire included the 12 questions that were used to measure the responses of the participants under three dimensions on a 9-point Likert scale (see Table 2).

The analysis of speech data is beyond the scope of this work and is analyzed in Offrede et al. (2023).

## 6 Results

As mentioned in Section 5.1, we used a WoZ approach to manage the robot’s turns. While the wizard was instructed to behave in the same way for both the conditions, we wanted to make sure that the wizard did not influence the turn taking of the robot, which could in turn influence the gaze aversion behavior of the participants. We calculated the turn gaps (time between when the participant had finished speaking and the robot started to speak) from the audio recordings of the interactions. A Mann-Whitney test indicated that there was no significant difference in turn gaps between condition *FG* (N = 249, M = 1.89, SD = ±2.67) and condition *GA* (N = 243, M = 1.77, SD = ±1.63), W = 29848, *p* = 0.797. This shows that the wizard managed the turns in the same way across conditions.

### 6.1 Effect of Robot’s gaze aversion behaviour

Of the 33 participants recorded, we excluded two participants’ data from the analysis as the gaze data was corrupted due to some technical problems with the eye-tracker. Additionally, gaze data from the eye-trackers were not always available for all timestamps, due to various reasons such as calibration strength and detection efficiency. When averting gaze, participants also moved their head away from their partner’s face. This varied a lot from participant to participant and led to instances where the robot’s face was out of the eye-tracker’s camera frame. Moreover, there were instances where Haar-cascade could not detect the robot’s face for some timestamps due to various reasons. These factors resulted in instances where it was not possible to determine if there was a gaze aversion or not. We were able to capture 87.38% of gaze data (data loss = 12.62%), which is normal for eye-trackers (Holmqvist, 2017). Overall, only 1.6% of the data (8170 timestamps out of 504011) was affected by the technical constraints which led to the exclusion of data. Thus, instances of gaze aversion by participants where Furhat was out-of-frame (due to head movement) are not very common. Also, the amount of data lost in this way was the same across conditions so we do not believe that the excluded data had any influence on the results reported.

**FIGURE 3:**
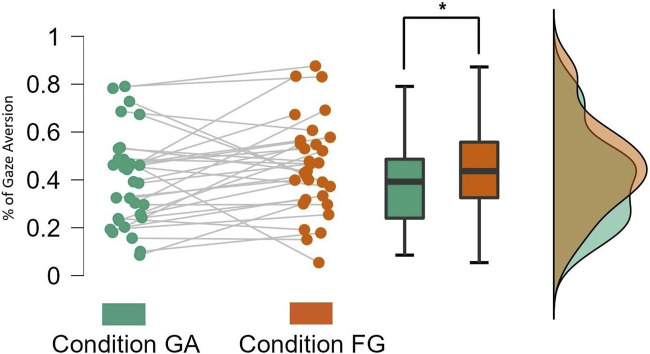
Total % of gaze aversion while participants were *Speaking*.

On average, participants averted their gaze more in the *FG* condition as compared to the *GA* condition when they were *Speaking*. A two-tailed Wilcoxon signed-rank test indicated a significant difference in gaze aversion across conditions when the participants were *Speaking* (*W* = 142.0, *p* = 0.037), as shown in Figure 3. This supported ***H1a***, which predicted that participants would avert their gaze for a longer duration when there is no gaze aversion by the robot (i.e., the *FG* condition). There was no significant difference between conditions when participants were *Listening* (*W* = 150.0, *p* = 0.194) which is expected (see 4). The mean values of gaze aversion when participants were *Speaking* and *Listening* can be found in Table 3.

**TABLE 3:** Mean % of gaze aversion per condition.

	Condition: GA		Condition: FG	
*ga%*	Mean	SD	Mean	SD
*Speaking*	0.399	±0.195	0.456	±0.199
*Listening*	0.112	±0.084	0.137	±0.155

**FIGURE 4:**
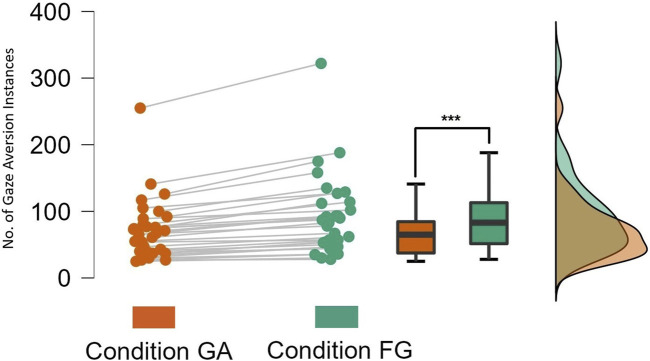
Number of Gaze Aversion instances while participants were *Speaking*.

Analyzing the number of gaze aversion instances performed by the participants while *Speaking* showed that participants looked away from the robot more frequently in the *FG* condition (Mean = 91.742, SD = ±60.158) as compared to the *GA* condition (Mean = 70.774, SD = ±46.141). As shown in Figure 4, a two-tailed Wilcoxon signed-rank test indicated a significant difference in the number of gaze aversion instances across conditions (*W* = 496.00, *p* < 0.001). This supported ***H1b***, which predicted that participants would look away from the robot more often when there is no gaze aversion by the robot. It can be seen that the effect of robot’s gaze aversion on participants’ gaze behavior is stronger and more distinct when analyzing gaze aversion instances. We argue that gaze aversion instance is a better metric to verify the effect.

### 6.2 Gaze aversion when participants were Speaking and Listening

It is already known from the HHI literature (Argyle and Cook, 1976; Cook, 1977; Ho et al., 2015) that people exhibit fewer gaze aversions while listening and more while speaking. We were interested to see if there was a similar pattern emerging from the data.

**FIGURE 5:**
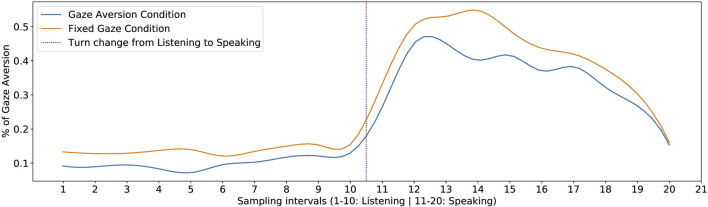
% of Gaze Aversion while participants were *Listening* and *Speaking*, for the two conditions.

To verify this, we first calculated the % of gaze aversion when participants were listening to and answering each of the robot’s questions. Since the durations of both *Speaking* and *Listening* varied from one participant to the other, we normalized the time into 10 intervals for *Speaking* and 10 intervals for *Listening* phase. Next, we found the aggregate % of gaze aversion for all the questions when *Speaking* and *Listening*. The resulting plot can be seen in Figure 5.

We can see a clear trend emerging from the plot with the low gaze aversion during the *Listening* phase when the participants listened to the robot. However, just before taking the floor (*Speaking* phase), it can be seen that the gaze aversion starts increasing. This is in line with the findings from Ho et al. (2015), who found that speakers usually started their turns with gaze aversion and averted their gaze before taking the turn. We also notice that the gaze aversion peaks at around 20%–30% of the speaker’s turn, before starting to fall. Towards the end of the turn, we see a sharp decline in gaze aversion. This is consistent with the findings from Ho et al. (2015), which show that people end their turns with their gaze directed at the listener. It can also be seen that even though the gaze aversion behavior of participants followed a similar pattern for both conditions, the amount of gaze aversion was lower for the *GA* condition. This further supports hypothesis **H1**.

### 6.3 Results from the questionnaire

On analyzing the responses from the questionnaire, all three dimensions were found to have good internal reliability (Cronbach’s *α* = 0.8, 0.71 & 0.92 respectively). The participants found the robot under the *FG* condition to be significantly more *Human-Like* (Student’s t-test, *p* = 0.029). This result was unexpected and is further discussed in Section 7. We did not find any significant differences for the other two dimensions. The mean score for the LexTALE test was 80.515% (SD = ±12.610) which showed that the participants had good English proficiency (Lemhöfer and Broersma, 2012).

### 6.4 Exploratory analysis: Topic intimacy

**FIGURE 6:**
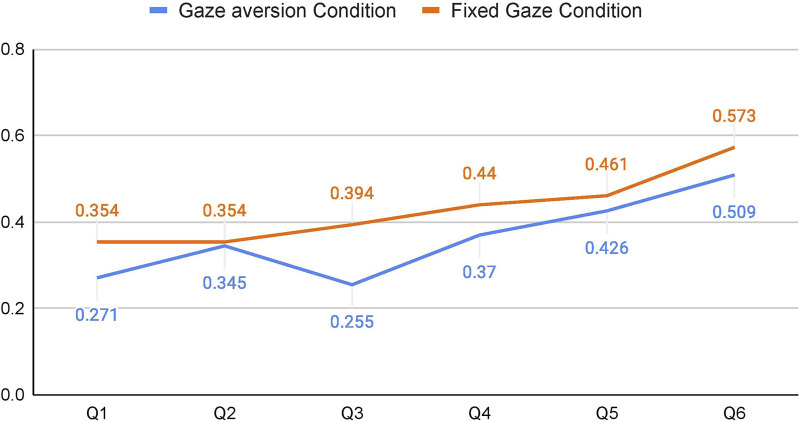
Mean Gaze Aversion per Question while participants were *Speaking*.

Apart from analyzing the data for **H1**, we were also interested in whether any trends emerged through an exploratory analysis of the topic intimacy of the questions and gaze aversion. By plotting the mean % of gaze aversion values of all participants for each question during the *Speaking* phase, we can see that there is an increase in gaze aversion as the intimacy values increase with the question order (cf. Figure 6).

We fit a GLMM (Generalized Linear Mixed Model) with mean gaze aversion values per question of each participant as the dependent variable (JASP Team, 2023). The questions’ order and the conditions were used as the fixed effects variables, and we included random intercepts for participants and random slopes for question order and condition per participant. The model suggested that the *gaze aversions increased as the intimacy values increased* (*χ* ^2^ = 41.32, *df* = 5, *p* < 0.001). It also suggested that there was more gaze aversion in the *FG* condition as compared to the *GA* condition (*χ* ^2^ = 4.244, *df* = 1, *p* = 0.039). There were no interaction effects observed. The coefficients of the model can be found in Table 4.

**TABLE 4:** Fixed effect estimates of the GLMM model.

Term	Estimate	SE	t
Intercept (Question 6)	0.396	0.030	13.138
Question 1	−0.084	0.022	−3.846
Question 2	−0.046	0.021	−2.224
Question 3	−0.071	0.019	−3.731
Question 4	0.009	0.018	−0.515
Question 5	0.047	0.023	2.085
Condition:*GA*	−0.033	0.016	−2.098

**FIGURE 7:**
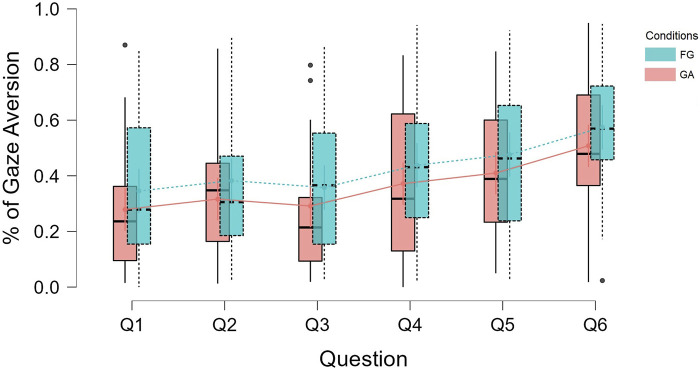
Distribution of mean gaze aversion per condition per question order.

This points in the direction of a positive correlation between topic intimacy and gaze aversion. One interpretation of this finding is that participants tend to compensate for the discomfort caused by highly intimate questions by averting their gaze. This is in line with previous findings from HHI that suggest that a change in any of the conversational dimensions like proximity, topic intimacy or smiling would be compensated by changing one’s behavior in other dimensions (Argyle and Dean, 1965). Figure 7 is a visualization of how gaze aversion varied for each condition under each question. It can be seen that the gaze aversion was higher for *FG* for all the questions (except Q3), and that there is an increase of gaze aversion with the increase in question number (which in turn is the topic intimacy value for the question).

The finding here is interesting because it could mean that the participants compensated for topic intimacy with gaze aversion even when it is a robot that was asking the questions. However, since we didn’t control for the order if the questions, this could also be because of other factors such as a cognitive effort and fatigue. Further studies should narrow down the factors that influenced such behavior.

## 7 Discussion

The results suggest that participants averted their gaze significantly more in the *FG* condition. Moreover, they had more gaze aversion instances in the *FG* condition. This was supported by both Wilcoxon signed-rank tests (see Section 6) and an exploratory GLMM (see Section 6.4). The results are in line with hypothesis **H1**: people compensate for the lack of robot gaze aversion by producing more gaze aversions themselves (Argyle and Dean, 1965; Abele, 1986).

We did not observe a significant difference across conditions in gaze aversion when participants were *Listening*. This could be attributed to the fact that there were too few gaze aversions during this phase to observe a significant difference, which is also suggested by prior studies in HHI (Ho et al., 2015). The gaze aversions varied between 11%–14%, which meant that the participants directed their gaze at the robot for about 86%–89% of the time. This is higher than the numbers reported in HHI, where listeners direct their gaze at speakers 30%–80% of the time (Kendon, 1967). Our findings coincide with the findings in Yu et al. (2012), where they reported that humans directed their gaze more at a robot than at another human.

Unexpectedly, participants rated the robot in the *FG* condition as more human-like compared to the *GA* condition. A key reason for that could be the way the *GA* interaction started. The GCS used would make the robot keep looking at random places in the environment unless the interaction is started by the researcher. This could have resulted in an unnatural behavior where the robot directs its gaze at random places even though the participant is already sitting in front of it. On the other hand, in the *FG* condition the robot kept on looking straight and only started to track the user when the interaction started. However, since the participant was sitting right in front of the robot, it would be perceived as the robot looking at the participant all the time.

While we did not find any significant differences in the other two dimensions (Conversation Flow & Overall Impression) assessed in the self-reported questionnaire, we did see a significant difference across conditions from the objective measures (i.e., gaze behavior). This could point to an effect that, even though it might not be explicitly perceived by people, a robot’s gaze behavior would implicitly affect human gaze behavior. This could also be an interesting direction for further study.

A further exploratory analysis of the data reveals a positive correlation between gaze aversion and topic intimacy of the questions. Thus, more intimate questions seem to lead to a larger avoidance of eye gaze. In our study, more intimate questions occurred towards the end of the conversation. As the order of the questions was fixed, the order may of course be a confounding factor. However, we are not aware of other work showing that humans would avoid eye gaze more and more over the conversation. We argue that eye gaze is rather related to the topic (intimacy), but further work is needed that controls for this potential confound.

## 8 Limitations and future work

The participants of our study had a rather large age span and we had only male participants. A clear limitation of this study is the lack of a balanced dataset. As the results obtained are only for male participants, these results do not necessarily generalize to other genders. The choice for male participants was methodologically and logistically motivated. Firstly, topic intimacy has been found to be perceived differently by people of different genders (Sprague, 1999). Thus, intimacy during the interaction might be affected by the participants’ and robot’s gender. To reduce the influence of this variable (given that it is not a variable of interest in this study), we controlled it by recruiting participants of only one gender.

In addition to the participants' gaze behavior, the recorded data were also used to analyze their speech acoustics in relation to that of the robot (Offrede et al., 2023). Since sex and gender are known to impact acoustic features of speech (Pépiot, 2014), all processing and analysis of data need to be carried out separately for males and females. This would reduce the statistical power of the acoustic analysis, leading us to choose participants from only one sex. Given the choice between female or male participants, males were chosen since they are more numerous in the institute where we collected data.

Further studies with a more diverse participant pool and female-presenting robots would be needed to verify this effect in general. However, it is interesting to note that a recent study (Acarturk et al., 2021) found no difference in gaze aversion behavior due to gender. The authors concluded that GA behavior was independent of gender and suggested “that it arises from the social context of the interaction.”

It is known that culture also influences our gaze behavior on many levels, such as how we look at faces (Blais et al., 2008) or interpretation of mutual gaze and gaze aversions (Collett, 1971; Argyle and Cook, 1976). McCarthy et al. (2006) observed that people’s mutual gaze and gaze aversion behaviors during thinking differed based on the culture of the individuals. However, recent studies have challenged some of aspects of cultural influences that have been reported previously (Haensel et al., 2022). Nonetheless, investigating any effect culture of participants may play on their gaze behavior when interacting with a robot could also be an interesting area to look into in the future.

## 9 Conclusion

In this paper, we investigated whether a robot’s gaze behavior can affect human gaze behavior during HRI. We conducted a within-subjects user study and recorded participants’ gaze data along with participants’ responses. The analysis of participants’ eye gaze in both conditions suggests that they tend to avert their gaze more in the absence of gaze aversions by a robot. An exploratory analysis of the data also indicated that more intimate questions may lead to a larger avoidance of mutual gaze. The existence of a direct relationship between robot’s gaze behavior and human gaze behavior is an original finding.

The study also shows the importance of modelling gaze aversions in HRI. In the absence of robot gaze aversions, the interaction may become more effortful for the user while trying to avoid frequent mutual gaze with the robot. These findings go hand in hand with the Equilibrium Theory suggesting a trade-off relation between the robot’s and user’s interactive gaze behavior. Our findings are helpful for designing systems more capable of adapting to the context and situation by taking human gaze behavior into account.

## Data Availability

The original contributions presented in the study are included in the article/Supplementary Material, further inquiries can be directed to the corresponding author.
